# Efficacy and safety of sodium-glucose cotransporter inhibitors in hypertrophic cardiomyopathy: a systematic review

**DOI:** 10.1016/j.ijcha.2026.101906

**Published:** 2026-03-30

**Authors:** Lorenzo Bianchi, Luisalice Afonso, Taras Chendey, Sravani Modumudi, Andrea Minghini, Job A.J. Verdonschot, Stephane R.B. Heymans

**Affiliations:** a Department of Cardiology, Cardiovascular Research Institute Maastricht (CARIM), Maastricht University Medical Center, Maastricht, the Netherlands; b European Reference Network for Rare, Low Prevalence, and Complex Diseases of the Heart, Amsterdam, the Netherlands; c Department of Medicine, Federal University of Cariri, Barbalha, Brazil; d Department of Hospital Therapy, Uzhhorod National University, Uzhhorod, Ukraine; e George Washington University School of Medicine and Health Sciences, Washington (DC), USA; f Department of Internal Medicine, University of Genova, Genova, Italy; g Department of Clinical Genetics, Maastricht University Medical Center, Maastricht, the Netherlands; h Department of Cardiovascular Sciences, Centre for Vascular and Molecular Biology, KU Leuven, Leuven, Belgium

**Keywords:** Hypertrophic cardiomyopathy, SGLT inhibitors, Heart failure, Systematic review

## Abstract

**Background:**

Sodium-Glucose Cotransporter inhibitors (SGLTi) are established therapies for heart failure (HF), chronic kidney disease, and type 2 diabetes. Their role in hypertrophic cardiomyopathy (HCM) remains undefined. This systematic review synthesizes available evidence on SGLTi efficacy and safety in HCM.

**Methods:**

We systematically searched PubMed, Embase, and Cochrane to 2 June 2025 for studies reporting cardiovascular outcomes or safety adverse events in HCM patients treated with SGLTi.

**Results:**

Three studies were included, comprising 5046 HCM patients, of whom 2523 (50%) received SGLTi. The population was predominantly male (68.9%), mean age 63.4 (±12.7), with high diabetes prevalence (90.6%). Cohorts were heterogeneous: one included 41.6% obstructive HCM, one only non-obstructive HCM, and one unspecified obstruction. Follow-up ranged 6–37.2 months. The high prevalence of diabetes and hypertension, along with limited genetic data, suggest some patients may have metabolic-mixed phenotypes rather than classic sarcomeric HCM.

Two studies, (n = 4998, 90.5% diabetics) reported an association between SGLTi and reduced all-cause mortality. HF hospitalizations results were inconsistent: one study (n = 4126, 100% diabetics) showed reduction, while two other (respectively n = 48, 100% diabetics, and n = 872, 45.5% diabetics) resulted neutral. Two studies (n = 920) reported comparable adverse events.

**Conclusion:**

Evidence on SGLTi in HCM is limited, largely observational, and confined to diabetic-metabolic patients. Observed reduction in all-cause mortality likely reflects these metabolic or mixed phenotypes rather than classical sarcomeric HCM. Effects on HF hospitalizations remain uncertain. No safety concerns emerged. These findings are hypothesis-generating and highlight need for randomized controlled trials of SGLTi in HCM with or without diabetes.

## Highlights

• Current evidence on SGLTi in HCM is limited and mostly from diabetic patients;
• Preliminary data suggest SGLTi may reduce mortality in mixed-metabolic HCM;
• Benefit seems related to metabolic or mixed phenotypes, rather than sarcomeric HCM;
• Data on heart failure hospitalizations and disease progression are inconsistent;
• Safety outcomes appear similar to controls, with no excess adverse events.

## Graphical abstract

**Figure:**
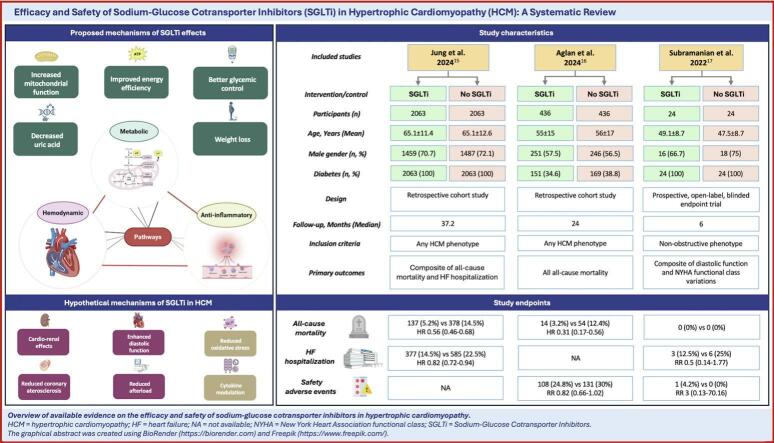

## 1 Introduction

Hypertrophic cardiomyopathy (HCM) is a genetically heterogeneous myocardial disorder characterized by left ventricular hypertrophy that cannot be explained by pressure overload, such as hypertension or valvular heart disease [1]. Affecting approximately 1 in every 500 individuals worldwide, [2] HCM is a major cause of heart failure (HF), stroke, arrhythmias, and sudden cardiac death [3], [4].

Current pharmacological treatment strategies for HCM primarily include beta-blockers, non-dihydropyridine calcium channel blockers, and disopyramide, [5] which mainly target symptom relief, especially in obstructive forms of HCM. More recently, myosin inhibitors such as mavacamten and aficamten have emerged as promising therapeutic options, demonstrating both clinical and quality-of-life benefits in patients with symptomatic obstructive phenotype [6], [7].

However, to date, no pharmacological therapy—whether for obstructive or non-obstructive forms—has been shown to confer prognostic benefit. In non-obstructive HCM, evidence for symptomatic improvement with medical therapy remains limited, further highlighting the need for therapies capable of modifying disease progression and improving long-term outcomes.

Sodium-glucose cotransporter (SGLT) inhibitors, initially developed for treating diabetes, have demonstrated consistent cardiovascular benefits in patients with HF, mediated by favourable effects on left ventricular systolic and diastolic function [8]. Beyond their cardioprotective role, SGLT inhibitors have also become a cornerstone in the management of chronic kidney disease and type 2 diabetes [9], [10].

Through hemodynamic, metabolic, and anti-inflammatory pathways, SGLT inhibitors exert pleiotropic effects that translate into meaningful reductions in cardiovascular outcomes. Their potential relevance to HCM lies particularly in improving left ventricular diastolic function, a hallmark abnormality in this condition [11], [12]. Anyway, because the pathophysiology of HCM—driven in part by genetic susceptibility—differs from that of ‘classical’ heart failure with preserved ejection fraction (HFpEF), which is largely associated with sex, age, and metabolic syndrome, it remains uncertain whether SGLT inhibition will also provide benefit in HCM patients.

To evaluate their potential role in this context, we conducted a systematic review of available evidence on efficacy and safety of SGLT inhibitors in patients with HCM, both with and without concomitant diabetes.

## 2 Methods

### 2.1 Study design

This systematic review was conducted in accordance with the Preferred Reporting Items for Systematic Reviews and Meta‐Analyses (PRISMA) guidelines [13]. The study protocol was registered within the PROSPERO International Prospective Register of Systematic Reviews with the following ID: CRD420251082029.

### 2.2 Ethics statement

As this is a systematic review based solely on published data, no ethical approval or informed consent from participants was required.

### 2.3 Data sources and search strategy

Databases included PubMed, Embase and Cochrane Library from inception to June 2, 2025.

The search strategy combined keywords and Medical Subject Headings (MeSH) terms related to HCM and SGLT inhibitors (*Supplementary Materials, Method S1*). Reference lists of eligible studies were also screened to identify additional records. The search was limited to articles published in English.

### 2.4 Eligibility criteria

Inclusion criteria were as follows:

1) observational studies and clinical trials involving adult patients (≥18 years) with HCM (diagnostic definition provided in *Supplementary Materials, Method S2*) treated with SGLT inhibitors compared to those not receiving SGLT inhibitors; ICD-based diagnoses, though less precise than systematic genetic or imaging-based diagnosis, were included to capture large real-world cohorts; their limitations were acknowledged in interpretating findings;
2) studies reporting at least one of the following outcomes:
  a. all-cause mortality, heart failure hospitalization, or heart failure exacerbation;
  b. prevalence of safety adverse events potentially related to SGLT inhibitors (including urinary tract infections, acute kidney injury, hypotension and syncope) between groups.

Exclusion criteria comprised conferences abstracts, case reports/series, reviews, preprints, preclinical studies, and non-English publications. The full list of inclusion and exclusion criteria is provided in the *Supplementary Materials, Method S3*.

### 2.5 Study selection and data collection process

Search results were screened using Rayyan reference manager software (Qatar Computing Research Institute, Doha, Qatar). After removal of duplicate records, two authors independently screened titles and abstracts for potentially relevant studies, followed by full-text review to confirm eligibility based on predefined inclusion criteria. Disagreements were resolved through consensus with a third author.

Extracted data included: study characteristics (name of the first author, year of publication, total number of participants, intervention, design and site study), baseline demographic and clinical characteristics of the patients (age, gender, HCM phenotype, genetic background, septal reduction therapies), cardiovascular comorbidities (hypertension, diabetes, atrial fibrillation), baseline left ventricular ejection fraction (LVEF), pharmacological treatments and medical devices (implantable cardioverter defibrillator or cardiac resynchronization therapy), duration of follow-up, efficacy outcomes (all-cause mortality, sudden cardiac death, HF hospitalization, HF exacerbations, ventricular arrythmias), safety outcomes (adverse events potentially related to SGLT inhibitors: acute renal failure, urinary tract infections, symptomatic hypotension and syncope).

### 2.6 Data synthesis and statistical analysis

Categorial variables are reported as counts and percentages; continuous variables as means ± standard deviations. When means and standard deviations were not directly reported, conversions were performed using established methods [14]. Hazard ratios (HRs) or risk ratios (RRs) with 95% confidence intervals (CIs) were used to summarize outcomes. If the HR or RR was not directly provided in a study, the RR was calculated from event counts and sample size. Absolute mean differences with 95% CIs were used to describe variations in continuous outcomes. Statistical analyses were performed using Review Manager (RevMan, Computer program, version 5.4. The Cochrane Collaboration, 2020).

### 2.7 Study risk for bias assessment

Risk of bias in non-randomized studies was independently assessed by two reviewers using the ROBINS-I tool (Risk Of Bias In Non-randomised Studies − of Interventions). Discrepancies were resolved through consultation with a third author. Each study was evaluated across seven domains: bias due to confounding, selection of participants, classification of interventions, deviations from intended interventions, missing data, outcome measurement, and selection of reported results. An overall risk of bias rating—low, moderate, or serious—was assigned to each study.

## 3 Results

### 3.1 Study selection

The initial database search identified a total of 440 records. After removing 37 duplicates, 403 unique records were screened based on title and abstract. Of these, 391 were excluded for not meeting the eligibility criteria. The full text of 12 articles was then assessed for eligibility, and three studies met the inclusion criteria and were included in the systematic review. A PRISMA flow diagram illustrating the study selection process is presented in *Supplementary Fig. 1*.

### 3.2 Study characteristics

All included studies were published within the last five years [15], [16], [17]. Two studies were conducted in Asia, [15], [17] and one was based on real-world data from a global federated registry (TrinetX Global Research Network) [16]. Study designs included one prospective, open-label, blinded endpoint trial, [17] and two retrospective analyses of national and international clinical dataset [15], [16]. Sample sizes ranged from 48 patients in the smallest study [17] to 4126 patients in the largest [15]. Characteristics of the included studies are summarized in Table 1*.*

**Table 1:** Characteristics of the included studies.

**Author**	**Year of publication**	**Total, n**	**Study intervention**	**Study site**	**Study design**	**Bias mitigation methods**
*Jung et al*. [15]	2024	4126	SGLTi vs. No SGLTi	Korea, National registry, multicentre	Retrospective cohort study	Propensity score matching
*Aglan et al*. [16]	2024	872	SGLTi vs. No SGLTi	International registry, multicentre	Retrospective cohort study	Propensity score matching
*Subramanian et al*[17]	2022	48	SGLTi vs. No SGLTi	India, single centre	Prospective, open-label, blinded endpoint trial	Blinded endpoint assessment

SGLTi = Sodium-glucose cotransporter inhibitors.

### 3.3 Baseline characteristics

The three studies included in the systematic review comprised a total of 5046 patients with HCM, of whom 2523 (50%) received SGLT inhibitors [15], [16], [17].

The analysed HCM population was heterogeneous: two studies included patients with any HCM phenotype, [15], [16] whereas one study enrolled exclusively individuals with the non-obstructive phenotype [17]. Overall, 68.9% of patients were male, and the mean age was 63.4 (±12.7) years. Diabetes was highly prevalent, affecting 90.6% of the overall study population. In two studies, all included patients had diabetes, [15], [17] whereas in the third study, 45.5% were affected [16]. Atrial fibrillation was reported in all three studies, with prevalences of 36.7%, 36,5% and 14.6%, respectively [15], [16], [17]. Chronic kidney disease was described in all the studies, affecting 12.9%, 16.4% and 18.8% of patients, [15], [16], [17] while body mass index was reported in two studies, with one study reporting a mean overweight phenotype, [17] and the other a mean obese phenotype (30.4 ± 7.2 and 27.2 ± 3.3) [16]. Hypertension was reported in only two studies, [15], [17] with prevalence rates of 96.5% in one cohort and 35.4% in the other. More detailed patient characteristics from the included studies are provided in Table 2*.*

**Table 2:** Baseline characteristics of the patients in the included studies.

	**Jung *et al.* 2024**[15]		**Aglan *et al.* 2024**[16]		**Subramanian *et al.* 2022**[17]	
	SGLTi	No SGLTi	SGLTi	No SGLTi	SGLTi	No SGLTi
Patients	2063 (50%)	2063 (50%)	436 (50%)	436 (50%)	24 (50%)	24 (50%)
Age (years)	65.1 ± 11.4	65.1 ± 12.6	56 ± 15	56.8 ± 17	49.1 ± 8.7	47.5 ± 8.7
Males, n (%)	1459 (70.7%)	1487 (72.1%)	255(58.5%)	231 (53%)	16 (66.7%)	18 (75%)
Obstructive HCM	NA	NA	185 (42.4%)	178 (40.8%)	0 (0%)	0 (0%)
Genetic P/LP variants	NA	NA	NA	NA	7 (29.2%)*	4 (16.7%)*
Diabetes	2603 (100%)	2603 (100%)	186 (42.7%)	211 (48.4%)	24 (100%)	24 (100%)
Atrial fibrillation	759 (36.8%)	756 (36.7%)	158 (36.2%)	160 (36.7%)	3 (12.5%)	4 (16.7%)
Hypertension	1992 (96.6%)	1989 (96.4%)	NA	NA	8 (33.3%)	9 (37.5%)
Kidney disease	261 (12.7%)	270 (13.1%)	72 (16.5%)	71 (16.3%)	4 (16.7%)	5 (20.8%)
BMI	NA	NA	30.5 ± 7.2	30.2 ± 7.1	26.8 ± 3.2	27.6 ± 3.4
LVEF	NA	NA	NA	NA	61.4 ± 7.2	62.5 ± 8.7
Concomitant HF medications − Beta-blockers − RAASi − MRA − Loop diuretics − CCB − Thiazide diuretics − GLP-1 Ra	1945 (94.3%) 1841 (89.2%) 801 (38.8%) 1163 (56.4%) 1489 (72.2%) 1352 (65.5%) 14 (0.7%)	1932 (93.7%) 1840 (89.2%) 809 (39.2%) 1156 (56.0%) 1516 (73.5%) 1335 (64.7%) 12 (0.6%)	334 (76.6%) 326 (74.8%) NA 287 (65.8%) NA NA	352 (80.7%) 324 (74.3%) NA 307 (70.4%) NA NA	22 (91.7%) 3 (12.5%) 16 (66.7%) 22 (91.7%) 2 (8.3%) 0 (0%) 0 (0%)	23 (95.8%) 3 (12.5%) 12 (50.0%) 24 (100.0%) 1 (4.2%%) 0 (0%) 0 (0%)
ICD/CRT-D	105 (5.1%)	98 (4.8%)	50 (11.5%)	26 (6%)	7 (29.2%)	5 (20.8%)
Septal myectomy	NA	NA	0 (0%)	30 (6.9%)	0 (0%)	0 (0%)

Categorial variables are reported as absolute value (%), and continuous variables as mean ± standard deviation.

† BMI was available for 152 patients in the SGLTi group and for 169 patients in the non SGLTi group. Additional data were retrieved via correspondence with the study authors.

### 3.4 Risk of bias

The risk of bias in the included studies, assessed using the ROBINS-I tool, is presented in *Supplementary Table S4*. Two studies were judged to have a moderate overall risk of bias, [16], [17] while one study was rated as serious, mainly due to confounding issues [15]. One study included all patients with diabetes and nearly all with hypertension (96.5%), representing a population with a metabolic-HCM phenotype [15]. This high prevalence of comorbidities may introduce serious bias in interpreting HCM outcomes, as some patients could have hypertensive heart disease or HF with preserved ejection fraction (HFpEF) rather than classical sarcomeric HCM. None of the included studies showed a low risk of bias, which is consistent with their non-randomized design. Moderate concerns were also noted across studies for outcome measurement and intervention classification, suggesting some limitations in the internal validity of the findings.

### 3.5 Study findings

The main findings of the included studies and outcomes are summarized in Table 3 and Table 4.

**Table 3:** Main findings of the included studies.

**Author**	**Total, n**	**Study intervention**	**Key inclusion criteria**	**Study duration**	**Primary outcomes**	**Secondary outcomes**
Jung *et al*. [15]	4126	SGLTi vs. No SGLTi	Any HCM phenotype	37.2 months	Composite of all-cause mortality and heart failure hospitalization	(1) all-cause mortality (2) heart failure hospitalization (3) Sudden cardiac death (4) ischaemic stroke
Aglan *et al*. [16]	872	SGLTi vs. No SGLTi	Any HCM phenotype	24 months†	All-cause mortality	(1) Acute heart failure exacerbation (2) all-cause hospitalization (3) cardiovascular symptoms* (4) potential SGLTi-related adverse events ††
Subramanian *et al.*[17]	48	SGLTi vs. No SGLTi	Non-obstructive phenotype	6 months^†^	Composite of diastolic function and NYHA class variations**	(1) Heart failure hospitalization (2) Systolic and diastolic function (3) NYHA functional class (4) 6MWT (5) NT-proBNP (6) adverse events ††

Follow-up duration is reported as median or as prespecified per protocol (†).

6MWT = 6-minute walk test; HCM = hypertrophic cardiomyopathy; NT-proBNP = N-terminal pro-B-type natriuretic peptide; NYHA = New York Heart Association; SCD = sudden cardiac death;

SGLTi = sodium-glucose cotransporter inhibitor.

infections were clearly reported, which we classified as safety events.

* Cardiovascular symptoms: chest pain, abnormal breathing, palpitations, and/or lower extremity oedema;

†† Potential SGLTi-related adverse events: defined by Aglan *et al*. [16] as hypotension, syncope, urinary tract infection, and acute renal failure; not explicitly defined by Subramanian *et al.,*[17] only urinary tract.

** Composite of diastolic function and NYHA class variations consisted of achieving both: improvement of at least 1.5 in the E/e’ ratio and a reduction of > 1 NYHA class.

**Table 4:** Main findings of the outcomes.

**Author**	**SGLTi**	**No SGLTi**	**All-cause mortality**	**Composite all-cause mortality and heart failure hospitalization**	**Heart failure hospitalization**	**Heart failure exacerbation**	**Safety adverse events**	**Main findings**	**Conclusion**
Jung *et al.*[15]	n = 2063	n = 2063	137 vs 378 (5.2% vs 14.5%) HR 0.56 (0.46–0.68)	429 vs 734 (16.5% vs 28.2%) HR 0.76 (0.67–0.86)	377 vs 585 (14.5% vs 22.5%) HR 0.82 (0.72–0.94)	NA	NA	Primary endpoint: reduced composite all-cause mortality-heart failure hospitalization Secondary endpoints: reduced all-cause death, heart failure hospitalization, SCD and ischemic stroke	Favourable for SGLT inhibitors in all the outcomes
Aglan *et al*. [16]	n = 436	n = 436	14 vs 54 (3.2% vs 12.4%) HR 0.31 (0.17–0.56)			51 vs 53 (11.7% vs 12.2%) RR 0.96 (0.67–1.38)	108 vs 131 (24.8% vs 30%) RR 0.82 (0.66–1.02)	Primary endpoint: reduced all-cause mortality Secondary endpoints: no difference in acute heart failure exacerbations; reduction in all-cause hospitalization, and in cardiovascular symptoms. No difference in potential SGLTi-related adverse events.	Favourable for SGLT inhibitors to reduce all-cause mortality and cardiovascular symptoms Neutral for heart failure exacerbation; Neutral for safety outcomes
Subramanian *et al.*[17]	n = 24	n = 24	0 vs 0(0% vs 0%)	NA	3 vs 6 (12.5% vs 25%)RR 0.5 (0.14–1.77)	NA	1 vs 0 (4.2% vs 0%) RR 3 (0.13–70.16)	Primary endpoint: improved composite diastolic function and NYHA class. Secondary endpoints: improved diastolic function, NYHA class, 6MWT and NTproBNP; no significant difference in heart failure hospitalization or systolic function; .No significant differences in urinary tract infections.	Favourable for SGLT inhibitors in clinical, echocardiographic, and laboratory parameters. Neutral in terms of heart failure hospitalization, and systolic function; Neutral safety profile, albeit limited number of events.

Categorial variables are reported as absolute value (%), with effect estimates (HR or RR and 95% CI).

Potential SGLTi-related adverse events: defined by Aglan *et al.*[16] as hypotension, syncope, urinary tract infection, and acute renal failure; not explicitly defined by Subramanian *et al.,*^17^ only urinary tract infections were clearly reported, which we classified as safety events.

NA = not available; SGLTi = sodium glucose cotransporter inhibitor.

Outcomes varied across studies, with all-cause mortality being the only endpoint consistently reported in all three studies. Two studies evaluated HF hospitalizations, [16], [17] whereas the third did not include this outcome, instead reporting HF exacerbation as a secondary outcome. [16], [17] Safety outcomes—adverse events potentially related to SGLT inhibitor therapy—were reported in two studies [16], [17]. In Subramanian *et al*.,[17] safety outcomes were not explicitly defined; only urinary tract infections were clearly reported, which we classified as safety events. Follow-up duration—median or mean depending on the study—ranged from 6 to 37.2 months [15], [16], [17].

A significant reduction in all-cause mortality was observed in the two largest studies:

Jung *et al*. (n = 4126) reported a 44% risk reduction (5.2% vs. 14.5%; HR 0.56; 95% CI 0.46–0.68; p < 0.01), [15] while Aglan *et al*. (n = 872) observed a 69% risk reduction (3.2% vs. 12.4%; HR 0.31; 95% CI 0.17–0.56; p < 0.001 [16]. In contrast, the smallest study (n = 48) reported no mortality events [17].

Of the two studies assessing HF hospitalizations, one found a significantly lower risk with SGLT inhibitor use (14.5% vs. 22.5%; HR 0.82; 95% CI 0.72–0.94; p = 0.004), [15] whereas the other reported no significant difference (12.5% vs. 25%; RR 0.50; 95% CI 0.14–1.77; p = 0.28) [17]. In Aglan *et al*., which assessed HF exacerbation rather than hospitalization for HF, rates were similar between patients treated with SGLT inhibitors and those not receiving SGLT inhibitors (11.7% vs. 12.2%; RR 0.96; 95% CI 0.67–1.38, p = 0.83) [16]. A detailed summary of the clinical outcomes with effect estimates is reported in the *Supplementary Table S1.*

Sudden cardiac death and ventricular arrhythmias were not consistently evaluated across the three studies. Only one study assessed sudden cardiac death, showing a favourable effect of SGLT inhibitors (1.4% vs 4.2%; HR 0.5; 95% CI 0.33–0.77; p = 0.001), [17] whereas two different study evaluated ventricular arrhythmias, observing comparable rates between groups (16.3% vs. 14.2%; RR 1.2; 95% CI 0.52–2.75; p = 0.4 in Aglan *et al*. and 4.2% vs. 4.2%; RR 1; 95% CI 0.89–1.13; p = 1 in Subramanian *et al*).[16], [17].

Soft outcomes—including clinical symptoms, functional parameters (New York Heart association [NYHA] class, six minute walk test [6MWT], LVEF, diastolic dysfunction), and laboratory biomarkers of HF (NT-proBNP concentrations)—were comprehensively assessed in only one study, [17] while another one evaluated only cardiovascular symptoms [16]. Subramanian *et al.* observed significant improvements in NYHA class, 6MWT performance, diastolic function, and NTproBNP concentration but no change in LVEF [17]. Similarly Aglan *et al.* reported a reduction in cardiovascular symptoms in SGLT inhibitor users [16]. A detailed summary of functional and laboratory outcomes with effect estimates is reported in *Supplementary Table S2.*

Regarding safety, adverse events potentially related to SGLT inhibitor therapy were generally neutral: in Aglan *et al*., the incidence was 24.8% vs. 30% (RR 0.82; 95% CI 0.66–1.02), [16] whereas in Subramanian *et al*. 4.2% vs. 0% (RR 3.00; 95% CI 0.13–70.16) [17]. Comprehensive data on safety outcomes, are reported in *Supplementary Table S3*.

## 4 Discussion

This systematic review is the first to evaluate the effects of SGLT inhibitors on cardiovascular outcomes and safety in patients with HCM. The three available studies comprised 5046 HCM patients, representing markedly heterogeneous populations, which is critical to consider when interpreting the findings.

In the largest—retrospective—cohort, [15] all patients had diabetes, and nearly all were hypertensive (96.5%), with substantial high use of antihypertensive therapies (72.8% on calcium channel blockers and 65.1% on thiazides diuretics). The observed prevalence of hypertension is markedly higher than in general or HCM population of comparable age, where rates typically range from 50 to 60% [18]. This suggests a population enriched with a metabolic-HCM phenotype in which HFpEF with hypertrophy may coexist. The absence of genetic data and the rely on ICD codes limits the reliability of the diagnosis of sarcomeric HCM, and raises the possibility that some patients classified as HCM may represent metabolic or mixed HFpEF phenotypes rather than classical sarcomeric HCM.

By contrast, the second retrospective study [16] did not report data on hypertension. Patients had elevated BMI (∼30 kg/m^2^) and obstructive HCM was present in 41.6% of patients, suggesting a population more consistent with classical sarcomeric HCM, albeit with a relevant metabolic burden.

The third study, [17] smaller in size, reported lower BMI values (∼27 kg/m^2^), no obstructive HCM, and partial genetic testing, identifying pathogenic or likely pathogenic variants in 29% of the SGLTi group and 17% of controls, providing the clearest evidence of genotype-positive sarcomeric HCM within these cohorts.

Taken together, these observations suggest that Jung *et al.*[15] mostly captured metabolic-HCM/HFpEF phenotypes, Aglan *et al.*[15] a mixed group including obstructive HCM enriched by metabolic comorbidities, and Subramanian *et al*.[17] a smaller but more classical sarcomeric HCM cohort. This heterogeneity likely explains some of the variability in outcomes and emphasizes the need for careful consideration of baseline comorbidities when interpreting the results.

Observed all-cause mortality rates in the non-SGLT inhibitor group was 12.4.% over 24 months of follow-up in the study by Aglan *et al*.,[16] and 14.5% over a median follow-up of 37.2 months in Jung *et al*. [15].

These rates are notably higher compared to those contemporary HCM cohorts: in the Share HCM registry (n = 4591), 8% of patients with any HCM phenotype died over a median follow-up of 34.8 months, [19] and in the MAVA-LTE (n = 231), 2.2% of patients with obstructive HCM treated with mavacamtem died over the median follow-up of 38.3 months [20]. This likely reflects the high cardiovascular risk profile of our analysed pooled population, particularly the predominance of diabetes.

The observed reduction in mortality associated with SGLT inhibitors is noteworthy; however, it likely reflects the predominance of metabolic or mixed phenotypes, labelled as HCM rather than classical sarcomeric phenotype. Supporting this interpretation, the magnitude of observed benefit exceeds that typically reported in randomized heart failure or diabetes trials, [8], [9], [10] suggesting possible confounding by indication, survivor bias, or unmeasured covariates.

Additional support for this hypothesis comes from the SCORED trial, which evaluated a population characterized by a high burden of metabolic comorbidity. All patients had diabetes and chronic kidney disease, the median body mass index was 31.8 kg/m^2^, and 31% had HF (15.8% with preserved EF, HFpEF). Although this population was not affected by HCM, its high-risk cardio-metabolic profile is similar to that of our cohort. In the SCORED, treatment with the dual SGLT inhibitor sotagliflozin significantly reduced the composite outcome of cardiovascular mortality and heart failure events (5.6 vs 7.5 events per 100 patient-years; HR 0.74; 95% CI 0.63–0.88; P < 0.001) [21]. Importantly, this benefit was consistent across all stages of heart failure (stage A, B and C/D), as well as in the population with HFpEF and HF with mildly reduced ejection fraction (HFmrEF) [21], [22]. Sotagliflozin also significantly reduced heart failure events (composite of heart failure hospitalizations and urgent visits: HR 0.67 (0.55–0.82), p < 0.001) and major adverse cardiovascular events (MACE: HR 0.77; 95% CI 0.65–0.91; p = 0.002) [21], [22], [23].

Taken together, evidence from the SCORED trial— although conducted in a population with cardiovascular comorbidities and not specifically in HCM—supports the concept that the clinical benefit of SGLT inhibition may be particularly pronounced in populations with a high burden of cardio-metabolic comorbidities, similar to that observed in our pooled HCM cohort.

Therefore, the mortality reduction in our pooled HCM population should not be interpreted as a class effect in sarcomeric HCM. Rather, it should be considered hypothesis-generating and plausibly driven by cardiometabolic risk reduction in populations enriched for diabetes and hypertension rather than a specific HCM mechanism of benefit.

Evidence on other cardiovascular outcomes, including HF hospitalization and sudden cardiac death, remains limited and inconsistent. Importantly, HF hospitalization is a relatively uncommon event in HCM and may not adequately capture clinically meaningful diseases progression, which in the HCM population is usually better reflected by symptom burden, functional capacity, LVOT gradients, diastolic dysfunction, and arrhythmic burden. Data on patient-centred outcomes in this analysis remain limited: in the study by Subramanian et al., treatment with SGLT inhibitors was associated with improvements in NYHA functional class, exercise capacity, diastolic function, and NT-proBNP concentrations, without significant changes in LVEF [17]. Although derived from a small cohort with short follow-up, these findings suggest that symptomatic, functional, and biomarker-based measures may represent more HCM-relevant indicators of treatment response than HF hospitalization alone.

From a physio-pathological standpoint, SGLT inhibitors confer cardiovascular protection in patients with diabetes through multiple pathways, including natriuretic/diuretic effects, improved cardiac energy efficiency via ketone utilization, modulation of mitochondrial metabolism, reduced sympathetic activation, and decreased inflammation and oxidative stress [24]. In HCM, independent of diabetes, potential benefits remain largely hypothetical. In vitro studies suggest that gliflozins may improve cardiac relaxation, calcium handling, cellular metabolism, and arrhythmogenesis [25]. Enhanced relaxation was observed in engineered human heart tissue carrying *MYH7*-R403Q7 or TNNT2-R92Q variants, [25] while murine HCM models exhibited reduced hypertrophy and fibrosis with empagliflozin [26].

Importantly, no safety concerns regarding SGLT inhibitors in HCM patients and concomitant diabetes were identified, as adverse event rates were comparable between treated and untreated groups [16], [17].

This finding is notable, as gliflozins, by increasing natriuresis, can lead to greater urinary output and, potentially favour volume depletion and worsening left ventricular outflow tract obstruction. Nevertheless, an excess of volume depletion has been observed only in chronic HF patients receiving furosemide-equivalent doses ≥ 40 mg [27]. Furthermore, the additive diuretic effect of SGLT inhibitors is more pronounced during the first days after initiation, [28] after which it is counterbalanced by adaptive mechanisms, including vasopressin-mediated urine concentration [29]. These safety observations are reassuring but largely apply to diabetic and non-obstructive HCM cohorts. Caution is warranted when extrapolating to patients with severe obstruction, high resting or provocable gradients, or on aggressive diuretic therapy, as risks of volume depletion, preload reduction, and LVOT obstruction cannot be excluded. While the reassuring signal is encouraging, small sample sizes and limited follow-up preclude definitive conclusions. Dedicated randomized clinical trials are warranted to further explore the effects of SGLT inhibitors in HCM patients with and without obstructive phenotype, and in genetic vs. gene-elusive patients. In addition, potential modifying factors, such as diabetes, hypertension, chronic kidney disease, and obesity-related phenotype, should be considered to better define the therapeutic value of these drugs across different disease subgroups.

Several trials are currently ongoing, with a stronger focus on patient-centred and HCM-specific outcomes rather than HF hospitalization. The SONATA-HCM (ClinicalTrials.gov ID NCT06481891), a randomized, double-blind, placebo-controlled trial, evaluates the efficacy of sotagliflozin on symptoms, functional capacity, and safety in obstructive and non-obstructive HCM;[30] the SOTA-CROSS HCM (ClinicalTrials.gov ID NCT06433050), a randomized, double-blind, crossover trial comparing sotagliflozin with placebo in non-obstructive HCM, assessing arrhythmic outcomes, intracavitary left ventricular (LV) gradients, functional capacity, systolic and diastolic function, NT-proBNP levels, and quality of life;[31] the ENDEAVOR-HCM (ClinicalTrials.gov ID NCT06580717) investigates the effect of enavogliflozin on diastolic function, biomarkers, exercise capacity, and arrhythmic burden in non-obstructive HCM [32]. Notably, though these trials are expected to provide clinically relevant informations, none of these trials address hard clinical outcomes such as all-cause mortality or HF hospitalization. Even if ongoing trials demonstrate symptomatic or functional benefit, hard outcome data will remain lacking, emphasizing the need to carefully contextualize the observed mortality reductions from current observational studies and underscores the importance of dedicated outcome-driven trials.

## 5 Limitations

Several limitations of our systematic review should be acknowledged.

First, the small number of included studies limits the generalizability of the findings across different clinical settings. To maximize study inclusion, we considered patients with any HCM phenotype and regardless of the specific indications for SGLT inhibitor therapy, such as diabetes, chronic kidney disease or HF. However, substantial heterogeneity in HCM phenotypes, inclusion criteria, intervention protocols, and reported outcomes precluded pooled analyses and limited quantitative interpretation.

Second, the two first studies were non-randomized, predominantly retrospective designs, which restricts causal inference due to residual confounding [15], [16].

Third, HCM diagnosis was often based on International Classification of Diseases and Related Health Problems (ICD-10) codes rather than clinical criteria, which may differ from routine clinical practice and limit the direct generalization of results.

Fourth, the predominance of patients with diabetes precludes the extrapolation of the observed effects to HCM patients without diabetes. In addition, the presence of mixed metabolic-hypertensive phenotypes raises the possibility that overlapping conditions such as hypertensive heart disease or HFpEF with hypertrophy were included, instead of classic sarcomeric HCM, which may have contributed to biased selection and outcome variability. Accordingly, the observed mortality benefit should be interpreted with adequate caution and can’t be generalized across all HCM populations and phenotypes.

Fifth, although available data suggest a reassuring safety profile in diabetic and non-obstructive HCM cohorts, the limited follow-up and small sample size preclude definitive or generalizable conclusions regarding safety. Caution is warranted in patients with significant obstruction or in those requiring intensive diuretic therapy.

Finally, limited data on genetic background of HCM precluded further evaluation of these important disease modifiers. This is particularly relevant given that metabolic traits such as obesity, hypertension, and diabetes are more prevalent in gene-negative HCM, suggesting that responses to SGLT inhibitors may differ across subgroups.

Taken together, these limitations underscore the need for genotype-stratified, prospective studies to confirm our findings and clarify the role of comorbidities in observed outcomes.

## 6 Conclusion

This systematic review evaluates SGLT inhibitors in patients with HCM. Evidence on SGLT inhibitors in HCM is currently limited and largely observational, with majority of data derived from patients with concomitant diabetes and/or hypertension. Current evidence suggests a potential reduction in all-cause mortality that likely reflects the presence of mixed-metabolic phenotypes, labelled as HCM, rather than classical sarcomeric disease, with diagnosis limited by reliance on ICD codes and incomplete genetic testing. Effects on HF hospitalization or HF exacerbation remain uncertain, with occasional signals of benefit. SGLT inhibitors showed a favourable safety profile, with no increase in adverse events reported. These findings are hypothesis-generating and highlight the urgent need for randomized controlled trials to confirm efficacy and safety, and to clarify the role of SGLT inhibitors in HCM patients both with and without diabetes, or other comorbidities.

## 7 Declaration of generative AI and AI-assisted technologies in the manuscript preparation process

During the preparation of this manuscript, the authors used ChatGPT to assist with sentence rephrasing.

All content was reviewed and edited by the authors, who take full responsibility for the published article.

## 8 Statement of authorship

LB takes responsibility for all aspects of the reliability and freedom from bias of the data presented and their discussed interpretation. LA, LB and TC designed the work. LA and TC performed the literature review. LA, LB, SM, AM, and TC wrote the paper. A.M designed and edited figures. JAJV and SRBH critically revised the work. We acknowledge that the authors used AI tool ChatGPT to assist with sentence rephrasing. All content was reviewed and edited by the authors, who take full responsibility for the published article. All authors have read and approved the final manuscript.

**Any potential conflicts of interest:** SH receives personal fees for independent scientific advice on early development in the field of heart failure for AstraZeneca, Ribocure, and CSL Behring, and receives research support from AstraZeneca and CSL Behring. JAJV received funding from the Dutch Heart Foundation (Dekker Clinical Scientist grant), the Dutch Research Council (Veni programme), and the academic funds of the Maastricht UMC + . JAJV is Associate Editor of the International Journal of Cardiology Heart & Vasculature.

## CRediT authorship contribution statement

**Lorenzo Bianchi:** Writing – original draft, Visualization, Validation, Supervision, Methodology, Formal analysis, Data curation, Conceptualization. **Luisalice Afonso:** Writing – original draft, Visualization, Formal analysis, Data curation, Conceptualization. **Taras Chendey:** Writing – original draft, Visualization, Validation, Methodology, Formal analysis, Data curation, Conceptualization. **Sravani Modumudi:** Writing – original draft, Visualization, Validation, Formal analysis, Data curation. **Andrea Minghini:** Validation, Software, Resources, Methodology. **Job A.J. Verdonschot:** Writing – review & editing, Validation, Supervision, Conceptualization. **Stephane R.B. Heymans:** Writing – review & editing, Visualization, Validation, Supervision, Conceptualization.

## Funding

None.

## Declaration of competing interest

The authors declare the following financial interests/personal relationships which may be considered as potential competing interests: [SH receives funding from the European Union Commission’s Seventh Framework programme under grant agreement N° 305507 (HOMAGE). We acknowledge the support of IMI2-CARDIATEAM, from the Innovative Medicines Initiative 2 Joint Undertaking (JU) under grant agreement N° 821508; The JU receives support from the European Union’s Horizon 2020 research and innovation programme and EFPIA; DCM NEXT, HORIZON-EIC-2022-PATHFINDERCHALLENGES 01 “CARDIOGENOMICS” from HORIZON European Innovation Council Grants/ European Commission, under grant agreement 101115416; grant of Dutch Research Council Open Call ZonMW-Metacor. Member of the European Reference Network for rare low prevalence and complex diseases of the heart-ERN GUARD-Heart. SH receives personal fees for independent scientific advice on early development in the field of heart failure for AstraZeneca, Ribocure, and CSL Behring, and receives research support from AstraZeneca and CSL Behring. JAJV received funding from the Dutch Heart Foundation (Dekker Clinical Scientist grant), the Dutch Research Council (Veni programme), and the academic funds of the Maastricht UMC+. JAJV is Associate Editor of the International Journal of Cardiology Heart & Vasculature. TC received speaker’s fees as well as travel and accommodation costs from AstraZeneca which is not related to the present work. Other authors have no conflicts of interest to declare related to this project. All authors have disclosed any actual or potential conflict of interest including any financial, personal or other relationships with other people or organizations within three years of beginning the submitted work that could inappropriately influence, or be perceived to influence, their work].
