# Supplementary material for: Efficacy and safety of sodium-glucose cotransporter inhibitors in hypertrophic cardiomyopathy: a systematic review
Source: Int J Cardiol Heart Vasc. 2026 Mar 30;64:101906. doi: 10.1016/j.ijcha.2026.101906 (PMC13068626; doi:10.1016/j.ijcha.2026.101906)
Supplement: Supplementary Data 1 [file mmc1.docx]

**Supplementary Materials

Supplemental Method S1.** **Full electronic search strategy**PUBMED: ("Sodium-glucose co-transporter 2 inhibitors" OR "Sodium-Glucose Transporter 2 Inhibitors"[Mesh] OR "SGLT2 inhibitors" OR SGLT2i OR "SGLT2 inhibitor" OR empagliflozin OR dapagliflozin OR canagliflozin OR ertugliflozin OR ipragliflozin OR luseogliflozin OR tofogliflozin OR "Sodium-glucose cotransporter 2 inhibitors") AND ("hypertrophic cardiomyopathy" OR "Cardiomyopathy, Hypertrophic"[Mesh] OR "HCM" OR "familial hypertrophic cardiomyopathy" OR "genetic cardiomyopathy")

EMBASE: ("Sodium-glucose co-transporter 2 inhibitors" OR "Sodium-Glucose Transporter 2 Inhibitors" OR "SGLT2 inhibitors" OR SGLT2i OR "SGLT2 inhibitor" OR empagliflozin OR dapagliflozin OR canagliflozin OR ertugliflozin OR ipragliflozin OR luseogliflozin OR tofogliflozin OR "Sodium-glucose cotransporter 2 inhibitors") AND ("hypertrophic cardiomyopathy" OR "Cardiomyopathy, Hypertrophic" OR "HCM" OR "familial hypertrophic cardiomyopathy" OR "genetic cardiomyopathy").

COCHRANE LIBRARY: ("Sodium-glucose co-transporter 2 inhibitors" OR "Sodium-Glucose Transporter 2 Inhibitors" OR "SGLT2 inhibitors" OR SGLT2i OR "SGLT2 inhibitor" OR empagliflozin OR dapagliflozin OR canagliflozin OR ertugliflozin OR ipragliflozin OR luseogliflozin OR tofogliflozin OR "Sodium-glucose cotransporter 2 inhibitors") AND ("hypertrophic cardiomyopathy" OR "Cardiomyopathy, Hypertrophic" OR "HCM" OR "familial hypertrophic cardiomyopathy" OR "genetic cardiomyopathy")

**Supplementary Method S2.** **Definitions of hypertrophic cardiomyopathy and outcomes**

Hypertrophic cardiomyopathy (HCM):

- Jung et al.^15^ defined HCM according to the International Classification of Diseases-10th Revision codes, including both obstructive and non-obstructive HCM (I42.1 or I42.2) and individuals who had HCM under the rare intractable diseases programme code (V127.1);
- Aglan et al.^16^ defined HCM according to the International Classification of Diseases-10th Revision codes, including both obstructive and non-obstructive HCM (I42.1 or I42.2).
- Subramanian et al.^17^ defined HCM in the presence of a clinical diagnosis of non-obstructive phenotype of HCM (left ventricular wall thickness >1.5 cm in the absence of abnormal loading conditions).
Importantly, the authors included only patients with a left ventricular ejection fraction (LVEF) >55%, symptomatic (New York Heart Association class >2 and an elevated N-terminal pro-B-type natriuretic peptide >300 pg/ml), with type 2 diabetes mellitus, and clinically stable on medical therapy for at least 3 months.

Cardiovascular symptoms: defined as chest pain, abnormal breathing, palpitations, and/or lower extremity oedema by Aglan et al.^16^

Safety adverse events: include potential sodium-glucose co-transporter inhibitors-related adverse events.
- Aglan et al.^16^ defined adverse events as hypotension, syncope, urinary tract infection, and acute renal failure;
- Subramanian et al.^17^ did not explicitly define adverse events, clearly reported only urinary tract infections, which we classified as safety adverse events.

Heart failure exacerbation: defined as acute systolic heart failure, acute on chronic systolic heart failure, acute diastolic heart failure, acute on chronic diastolic heart failure, acute combined systolic and diastolic heart failure, acute on chronic combined systolic and diastolic heart failure, according to International Classification of Diseases-10th Revision codes (I50.21, I50.23, I50.31, I50.33, I50.41, I50.43), by Aglan et al.^16^

**Supplemental Method S3.** **Inclusion and exclusion criteria**
Inclusion criteria were as follows:

1. Observational studies and clinical trials describing adult patients (≥18 years) with hypertrophic cardiomyopathy who underwent a treatment period with sodium-glucose co-transporter (SGLT) inhibitors;
2. Studies reported data about at least one of the following endpoints:
   a) the occurrence of all-cause mortality, or heart failure exacerbation in both treatment and control groups;
   b) the cumulative prevalence of adverse events between the groups.

Exclusion criteria were as follows:

1. Previous treatment with SGLT inhibitors
2. Studies that did not report outcomes of interest;
3. Studies with overlapping populations;
4. Studies for which the full text was not available;
5. Language different from English;
6. Conference abstracts;
7. Case reports and case series;
8. Narrative reviews;
9. Editorials, commentaries, and expert opinions;
10. Clinical practice guidelines, protocols, and dissertations.

**Supplementary Figure 1. PRISMA flow diagram of study screening and selection**


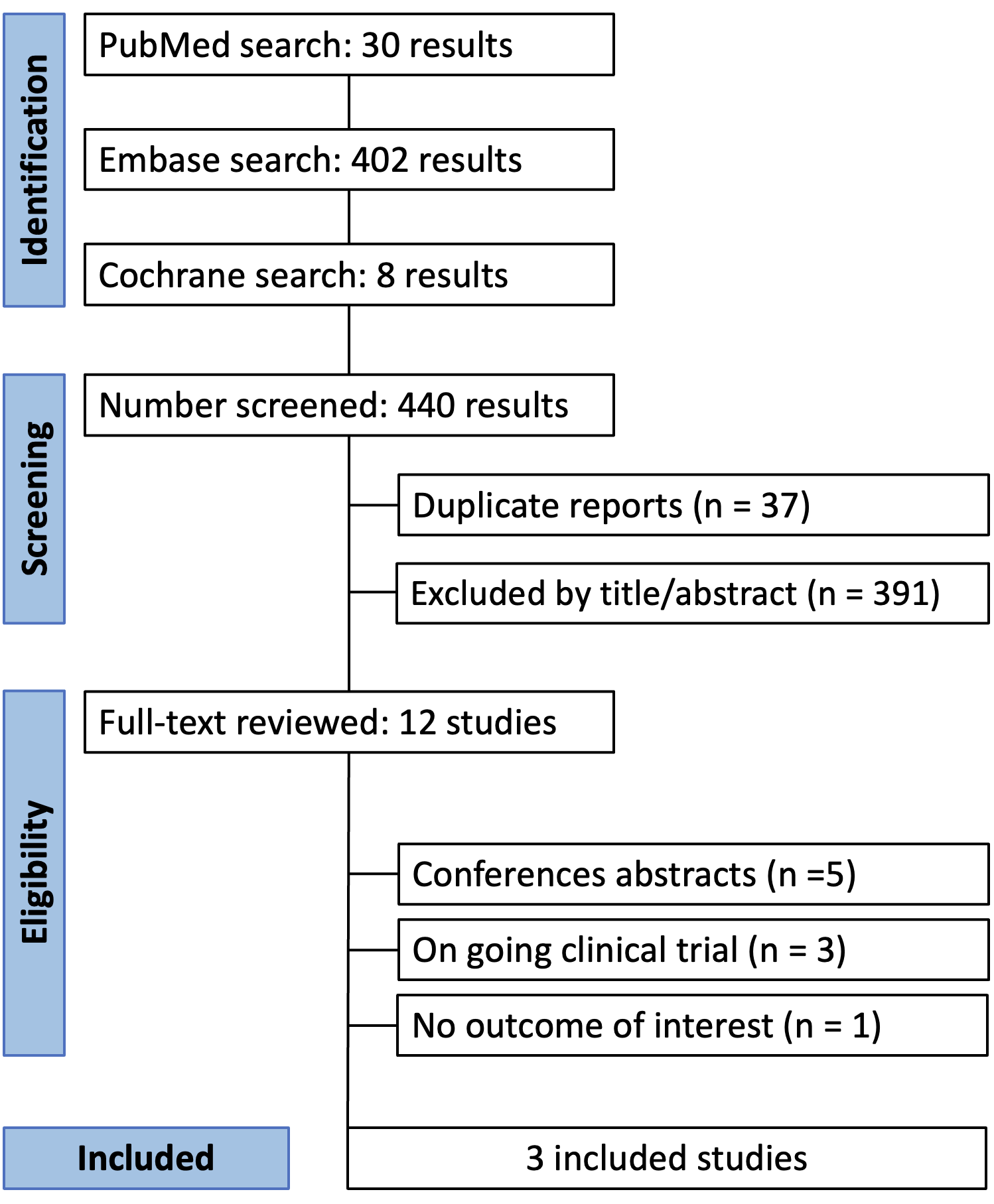


**Supplementary Table 1.** **Secondary clinical outcomes**

| Author | Jung *et al*.^15^ | | Aglan *et al*.^16^ | | Subramanian *et al*.^17^ | |
| --- | --- | --- | --- | --- | --- | --- |
| Groups | SGLTi | No SGLTi | SGLTi | No SGLTi | SGLTi | No SGLTi |
| Patients | n = 2063 | n = 2063 | n = 436 | n = 436 | n = 24 | n = 24 |
| Ischemic  stroke | 218 vs. 336 10.6% vs. 16.3% HR 0.738 (0.62-0.88) | | NA | NA | NA | NA |
| All-cause  hospitalization | NA | NA | 129 vs. 165 29.6% vs. 37.8% RR 0.78 (0.65-0.94) | | NA | NA |
| Sudden cardiac  death | 29 vs. 86 1.1% vs. 3.3% HR 0.5 (0.33–0.77) | | NA | NA | NA | NA |
| Ventricular  arrhythmias | NA | NA | 71 vs. 62  16.3% vs. 14.2% RR 1.2 (0.84-1.57) | | 1 vs. 1 4.2% vs. 4.2%  RR 1 (0.89-1.13) | |
| Cardiovascular  symptoms | NA | NA | 133 vs. 181 30.5% vs. 41.4% RR 0.73 (0.61-0.88) | | NA | NA |

Categorial variables are reported as absolute value (%), with effect estimates (HR or RR and 95% CI).
SGLTi = Sodium-glucose cotransporter inhibitor

**Supplementary Table 2.** **Secondary functional and laboratory outcomes**

| Author | Jung *et al*.^15^ | | Aglan *et al*.^16^ | | Subramanian *et al*.^17^ | |
| --- | --- | --- | --- | --- | --- | --- |
| Groups | SGLTi | No SGLTi | SGLTi | No SGLTi | SGLTi | No SGLTi |
| Patients | n = 2063 | n = 2063 | n = 436 | n = 436 | n = 24 | n = 24 |
| NYHA class  change | NA | NA | NA | NA | -0.4±0.2 | 0.1±0.2 |
| LVEF  change | NA | NA | NA | NA | 0.8±1.3 | 1.4±1.2 |
| Diastolic function  degree change | NA | NA | NA | NA | -0.3±0.06 0.1± 0.03 -0.4 (-0.5 to -0.3) | |
| E/e’ change | NA | NA | NA | NA | -3.0±0.9 1.1±1.2 -4.1 (-5.1 to -3.0) | |
| 6MWT  change | NA | NA | NA | NA | 47.9±21.2 6.6±20.6 54.5 (48.7 to 60.3) | |
| NTproBNP  change | NA | NA | NA | NA | -40.5±23.4 8.3±24.5 48.8 (44.4 to 53.1) | |

Categorical variables are presented as absolute values; continuous variables as mean ± standard deviation, absolute mean difference and 95% CI between groups.

LVEF = left ventricular ejection fraction; NYHA = New York Heart Association; NTproBNP = N-terminal pro-B-type natriuretic
peptide; SGLTi = Sodium-glucose cotransporter inhibitor; 6MWT = six-minute-walk test.

**Supplementary Table 3. Safety adverse events**

|  | SGLTi | No SGLTi |
| --- | --- | --- |
| Aglan *et al*.^16^ | n = 436 | n = 436 |
| Acute renal failure | 37 | 51 |
| Hypotension | 27 | 31 |
| Syncope | 27 | 21 |
| Urinary tract infections | 17 | 28 |
| Subramanian *et al.*^17^ | n = 24 | n = 24 |
| Urinary tract infections | 1 | 0 |
| Total | 109 | 131 |

SGLTi = Sodium-glucose cotransporter inhibitor

**Supplementary Table 4.** **Quality assessment and risk of bias summary for non-randomized studies (ROBINS-I)**

| Study | Bias due to confounding | Bias in selection of participants | Bias in classification of interventions | Bias due to deviations from intended interventions | Bias due to missing data | Bias in measurement  of outcomes | Bias in selection  of the reported result | Overall risk  of bias  judgement |
| --- | --- | --- | --- | --- | --- | --- | --- | --- |
| Jung *et al*.^15^ | Serious | Low | Moderate | Moderate | Low | Moderate | Low | Serious |
| Aglan *et al*.^16^ | Moderate | Low | Moderate | Moderate | Low | Moderate | Low | Moderate |
| Subramanian   *et al.*^17^ | Moderate | Moderate | Moderate | Low | Moderate | Moderate | Moderate | Moderate |
